# Inversion of the Uterus Successfully Reduced

**Published:** 1860-02

**Authors:** 


### INVERSION OF THE UTERUS SUCCESSFULLY REDUCED.

---

The following case of the successful treatment of inversion of the uterus, was reported for the *North Amer. Medico Chirurgical Review*, by Dr. Wm. Irvin, of Breakneck, Butler county, Pennsylvania.

"On the 26th of November, 1858, I was called to see Mrs. W., forty-eight years of age, in labor with her eleventh child. She was a large healthy woman, with an exceedingly roomy pelvis and pendulous abdomen. I found the os soft and dilated to the size of a dollar, but the pains were not strong for about six hours, when two or three uterine contractions were sufficient to expel the child, at the same time inverting the uterus with the placenta adherent. The cord being very short the weight of the child dragged down the organ between the thighs nearly as far as the knees.

"I severed the cord, and made several unsuccessful attempts to reduce the uterus, with the placenta still adhering. The hæmorrhage became very alarming, and the woman was sinking, when I remembered Professor Meigs's injunction to remove the placenta and return the organ by pressure upon its fundus. I therefore detached it as quickly as possible, and had the satisfaction of finding that the bleeding was much diminished.

"Placing my right hand under the uterus, and supporting it in a line with the axis of the pelvis with my left, I pressed the tips of my fingers firmly against the fundus and pushed it upward. After a little while the tumor softened, and I found I had indented the fundus, the pressure being continued until my fingers were arrested by the contracted os, which yielded in about one minute. I then carried my hand upward in the pelvic axis, and when I had introduced my arm, half way to the elbow, the fundus suddenly shot away from my hand, and the organ resumed its natural position. In a few minutes a contraction came on, and the organ returned to its proper situation.

"I watched the patient four hours, enjoining strict recumbency and rest. The case afterwards proceeded as usual, and the woman made a speedy recovery."

---

### GLYCERINE OINTMENT FOR ITCH.

[From the London Lancet.]

M. Bourguignon, so well known in Paris by his successful researches on "the acarus scabiei," has published in the *Gazette Medicale* the following formula. One general friction, not preceded by soap ablutions, is sufficient:—Yelks of two eggs; essence of lavender, lemon, and mint, of each seventy-five drops; essence of cloves and cinnamon, of each 120 drops; gum tragacanth, half a drachm; well-pounded sulphur, twenty-six drachms; glycerine, thirty-two drachms. Total weight, nearly eleven ounces. Mix the essences with the yelks of egg, add the gum tragacanth, make a good mucilage, and then add very gradually the glycerine and sulphur.

Many cures have been obtained by this preparation, which has the advantage of giving no pain.

The well-known Helmerich ointment being really useful, M. Bourguignon has modified it, and substituted glycerine for the axunge. In the altered form, the preparation is not any dearer, as efficacious, and less painful than the original ointment. It does not grease the clothes, and has an agreeable perfume.

Gum tragacanth, fifteen grains; carbonate of potash, thirteen drachms: well-pounded sulphur, twenty-six drachms; glycerine, fifty-two drachms; essence of lavender, lemon, mint, cloves, and cinnamon, each fifteen drops. Total weight nearly eleven ounces. Make a mucilage with the gum and one ounce of glycerine, add the carbonate, mix until it is dissolved, and then gradually add the sulphur and glycerine;—lastly, pour in the essences. With this compound, M. Bourguignon advises two general frictions of half an hour, within twelve hours of each other, and followed, twenty-four hours afterward, by a simple warm bath, as the glycerine is soluble in water. Two-thirds of the preparation should be used for the first friction, and the other third for the second.

